# Depopulation or vaccination? Tackling the COVID-19 crisis in prisons in Africa

**DOI:** 10.1186/s40352-022-00176-8

**Published:** 2022-03-05

**Authors:** Daniel Katey, Kabila Abass, Emmanuel Kofi Garsonu, Razak M. Gyasi

**Affiliations:** 1 Department of Geography and Rural Development, Kwame Nkrumah University of Science and Technology, Kumasi, Ghana; 2 African Population and Health Research Centre, Nairobi, Kenya

**Keywords:** Depopulation, Vaccination, Prison, Africa, COVID-19, Correctional facilities, Global public health, Prisoners, Coronavirus disease, Penitentiary staff and workers

## Abstract

Several attempts have been made by the global public health efforts and national governments to contain the spread and vulnerabilities to COVID-19. Evidence, however, shows a disproportionate upsurge in COVID-19 cases in correctional facilities such as prisons, particularly, in low- and middle-income countries (LMICs). The poor adherence to COVID-19 preventive protocols in these settings has raised a serious “moral panic” among the public. There are public health concerns about the most effective and state-of-the-art approach to tackling the continuous spread of the virus in prisons. This paper discusses the feasibility of depopulation and vaccination rollouts in combating COVID-19 in correctional facilities with a particular focus on African prisons. The paper proposes selective and strategic depopulation of prisoners, effective prioritization of vaccination among prisoners, and general sensitization of prisoners toward vaccine disbursement in this total institution.

Like many other total institutional settings, prisons have remained key hotspots for the spread of the novel coronavirus disease (COVID-19) since its declaration as a *Public Health Emergency of International Concern* (WHO, 2020; Cingolani et al., 2020; Pettus-Davis et al., 2021). Prisoners have been recognized as more susceptible to the virus compared to the general population (Maycock & Dickson, 2021; Mhlanda-Gunda et al. 2022). Similarly, Braithwaite et al. (2021) explain that overcrowding in prisons coupled with the convoluted human communication patterns in prison facilities have accounted for the spike in the prevalence of COVID-19 in such settings globally. COVID-19 has been assumed by many to be a massive public health concern in prisons in LMICs. However, Braithwaite et al. (2021) has indicated the dreadful effects of COVID-19 with dire public health implications in advanced country prisons. The impact of COVID-19 on prisoners, therefore, presents a global outlook. This is, at least, in parts, due to the lack of proactive governmental interventions to deal with the pandemic and the naked neglect of this special group in these unprecedented times in many countries, especially in LMICs (Xinhua, 2021).

Prisoners form a greater proportion of the marginalized groups that are easily forgotten by the policymakers (Franco-Paredes et al., 2020; Katey et al., 2021). The United Nations Office on Drugs and Crime (UNODC) has, therefore, been very categorical on the need for all UN member countries to incorporate prisons, prisoners, and prison workers into COVID-19 public health responses (Reuters, 2021). However, lack of compliance and privation of thorough preparedness on the path of most countries, have resulted in unprecedented death cases in prisons across the globe (Franco-paredes et al., 2020; Maycock & Dickson, 2021). For instance, according to The Marshall Project (2021), about 275,000 prisoners have contracted the virus in the United States, with over 1700 of them losing their lives. They further noted that the rate at which prisoners were contracting the virus was three times higher than in the United States general population. In Italy, Brazil, and India reports show the malicious scenes created by COVID-19 in prisons and other correctional facilities (Cingolani et al., 2020; Siva, 2020).

Reports on COVID-19 in Africa have not been different from those at the global level (Gyasi, 2020). In Africa, COVID-19 has been projected to claim over 300,000 lives, many of whom will be prison inmates and those in other correctional facilities (Reuters, 2021). In that regard, the United Nations Economic Commission for Africa (UNECA) noted that a sum of $100 billion would be needed to immediately resource a health and social safety net response (Reuters, 2021). This calls for immediate interventions from all stakeholders to strengthen their local and international COVID-19 response mechanisms particularly for those in incarceration (WHO, 2020). As a way of managing the menace in prisons, some African countries including Ghana, Egypt, and South Africa, have banned the intrusion of unauthorized people into prisons and other correctional facilities (Hamann et al., 2020; Muntingh, 2020; UNICEF, 2020). Similarly, following the WHO’s directives for prisons to be depopulated, several countries have released some prisoners to allow effective practices of the COVID-19 countermeasures such as social distancing. For instance, as of 24 March 2021, South Africa had released a total of 6128 prisoners and about 19,000 inmates had received bails (Xinhua, 2021). Moreover, the Ethiopian government released over 40,000 prisoners as a result of the exponential spread of the COVID-19 in their prisons (Xinhua, 2021). Again, Angola, Tunisia, Morocco, Egypt, Nigeria, Ghana, Kenya, and Zimbabwe have all made strides to release some prisoners to manage COVID-19 within their geo-borders (African Centre for the Constructive Resolution of Disputes [ACCORD], 2021).

Despite these significant policy advancements, COVID-19 continues to show its ugly face to many prison settings in Africa and globally (Hamann et al., 2020; Rutayisire et al., 2020). Anecdotal evidence demonstrates that the COVID-19-related death rate for Africa rose from 2.1% in July 2020 to 2.6% in February 2021 (BBC News, 2021a). Even though there have been concerns about the spread of the new variant on the continent, there is no evidence to suggest its impact on African prisons and correctional facilities (BBC News, 2021a). Whereas some scholars and renowned organizations have accepted depopulation as an effective tool to combat COVID-19 (Barsky et al., 2021; Braithwaite et al., 2021), others perceive the measure as a threat and an attempt to put the general population into jeopardy (Cingolani et al., 2020). Musawenkosi (2021) also criticizes prisons depopulation as highly unsustainable, that the approach can only result in managing the menace in prisons at the expense of the larger society.

The emergence of the COVID-19 vaccines stirred up controversies around the world over which category of people should be given early priority (Sallam, 2021). Several concerns were raised about the potential neglect of prisoners particularly among those in Africa. For instance, Ghana’s approach to distributing the first COVID-19 vaccine consignment focused extensively on the frontline COVID-19 workers including penitentiary staff, to the neglect of those incarcerated (Africa News, 2021). Similar trends were identified in South Africa, Cote d’Ivoire, among others (BBC News, 2021b; Otitoloju et al., 2020). The debate stirred much fear in prisoners and their families on the potential neglect of prisoners in the distribution of the vaccines (Barsky et al., 2021; Braithwaite et al., 2021). BBC News (2021b) further noted that the shortage of vaccines in most sub-Saharan African countries, taking into consideration the existing poor state of prisons has left many people wondering about the fate of the incarcerated, amidst rising death incidents in prisons.

The COVID-19 vaccination reports from some African countries indicate that the vaccines rollouts have led to a significant decrease in the compliance rate to the COVID-19 countermeasures and preventive protocols (Amoah & Simpeh, 2020; Giandhari et al., 2021). However, recently published literature indicates a strict adherence to the protocols in some prisons in South Africa (Giandhari et al., 2021). This dichotomy has created a dilemma in the minds of key stakeholders with some people questioning whether releasing prisoners to take full responsibility for their health or keeping them incarcerated while providing them with primary health care is the best approach to managing the menace in prisons (Barsky et al., 2021). Among the African general population, Afolabi & Ilesanmi (2021) suggests that prisoners may be highly hesitant to the vaccines due to negative belief systems and misconceptions about the COVID-19 vaccine. According to the WHO Africa (2021), only about 8% of Africans are fully vaccinated, compared to over 60% vaccination rate in high-income countries. This presupposes that the acceptance rate of the vaccine among prisoners is also likely to be low if no effective education and sensitization strategies are rolled out to reorient prisoners. Hence, we recommend an intensified mass education and sensitization of the population, particularly in rural areas with culturally-oriented modes (Edward et al., 2022). This should be done in partnership with local chiefs, religious leaders, and community health workers to constantly inform the populace about the importance of the vaccination in the prevention of the coronavirus vis-à-vis misconceptions about the long-term adverse effects of the vaccination.

Furthermore, it has been shown that the AstraZeneca vaccine (WHO recommended vaccine for low-income countries) is only 76% efficacious. Therefore, there is, but only a little guarantee for absolute immunity against the Coronavirus if all hope is banked on vaccination (WHO, 2021). We further propose that effective implementation of both depopulation and vaccination to sustainably curtail the spread of the virus in prisons will be eminent. Prisoners who are assessed as medically less active, frail, and vulnerable older adults and those nearing sentence completion should be considered for release. The incarcerated who are at very low risk of committing any serious crime (per the terms of their sentences) should also be considered in the depopulation exercise. A meticulous execution of selective and strategic depopulation alongside other robust measures outlined in the WHO Health in Prisons directives may unleash ample spaces in prison facilities. Very importantly, this will enhance the practice and adherence to social distancing protocols among the incarcerated as well as the penitentiary staff and workers. Our proposition of depopulation and vaccination as a strategic tool for managing COVID-19 in African prisons supports Barsky et al.’s (2021) recommendation for managing the disease in U.S prisons.

More so, medical units in these facilities should be equipped with well-trained medical practitioners and with sufficient COVID-19 safety kits and vaccines, to ensure a more formidable approach to combating the COVID-19 menace in prisons in LMICs and globally. Retained prisoners should be given equal COVID-19 vaccination preferences as the larger society after the depopulation exercise.

Furthermore, community re-entry plans should be developed by prison or correctional officials in conjunction with community-based programs, to incorporate a wide range of services, including housing, income support, health care (including COVID-19 vaccination), and COVID-19-related education. A well-planned implementation of measures can cushion the incarcerated and render them less vulnerable to the pandemic and at the same time protect the society from negative externalities of the depopulation exercise.

The fundamental human right of “good health” is a universal entitlement to all, irrespective of personality and geographical location. Prisoners, therefore, should be granted equal public health attention as the general population during COVID-19 and beyond to enhance their health and well-being.

## Data Availability

Not applicable.
